# Supplementary material for: Exploring Virus–Host Interactions Through Combined Proteomic Approaches Identifies BANF1 as a New Essential Factor for African Swine Fever Virus
Source: Mol Cell Proteomics. 2025 Jul 22;24(9):101038. doi: 10.1016/j.mcpro.2025.101038 (PMC12398270; doi:10.1016/j.mcpro.2025.101038)
Supplement: Supplemental Table S2 [file mmc2.docx]

**Supplementary material for Exploring virus-host interactions through combined proteomic approaches identifies BANF1 as a new essential factor for African Swine Fever Virus**

**Authors:** Juliette Dupré^1,2†^, Katarzyna Magdalena Dolata^3,*†^, Gang Pei^4^, Aidin Molouki^5^, Lynnette C Goatley^5^, Richard Küchler^3^, Timothy K Soh^6^, Jens B Bosse^6^, Aurore Fablet^1^, Mireille Le Dimna^2^, Grégory Karadjian^7^, Edouard Hirchaud^8^, Christopher L Netherton^5^, Linda K Dixon^5^, Ana Luisa Reis^5^, Damien Vitour^1^, Marie-Frédérique Le Potier^2^, Axel Karger^3,*†^ and Grégory Caignard^1,*†^


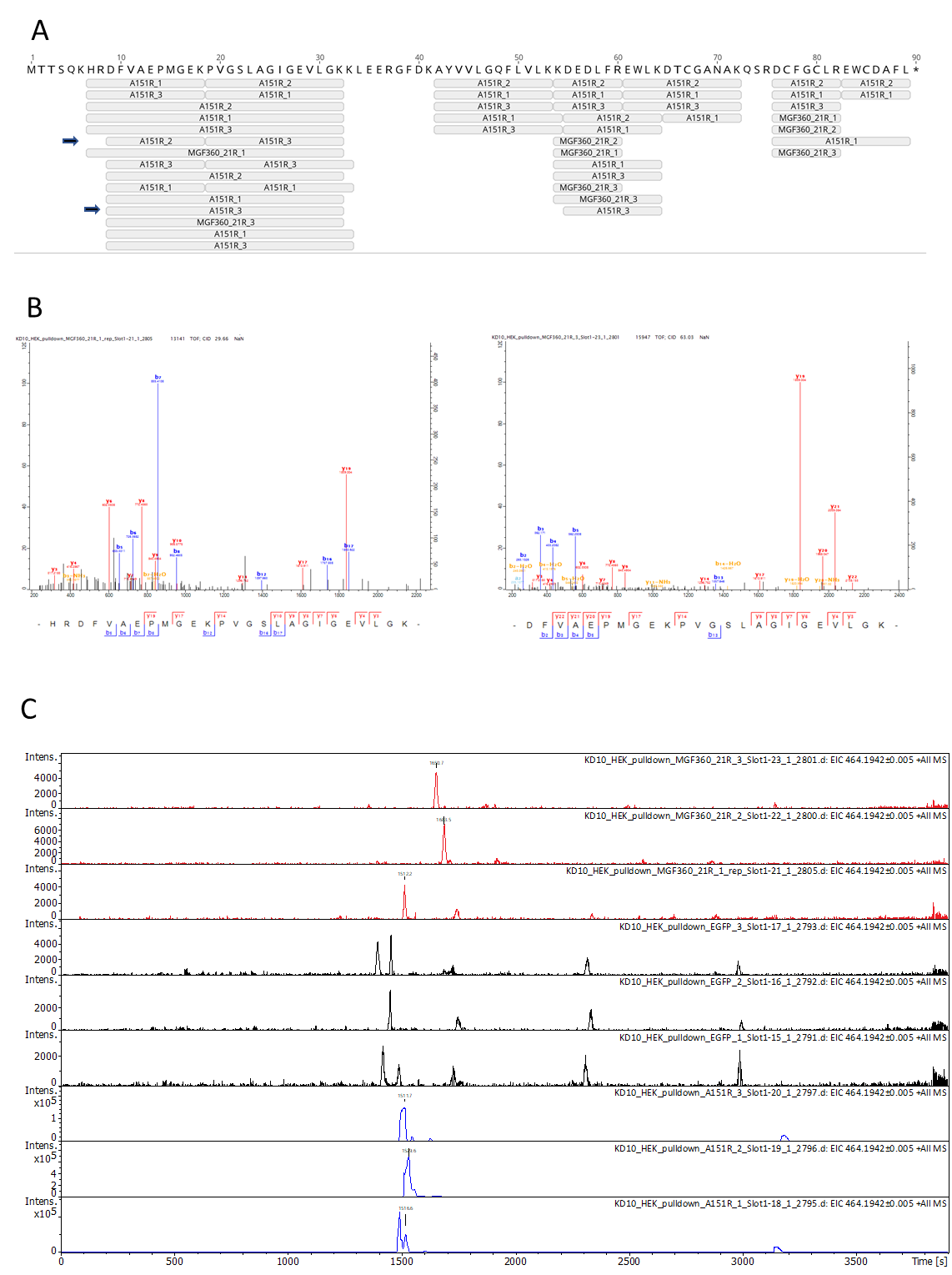


**Supplementary Figure 1.** **Peptide coverage and identification of BANF1 in AP-MS experiments with A151R and MGF360-21R.** (A) Coverages of the BANF1 sequence by tryptic peptides from the AP-MS analysis which was constructed on the basis of the data from the Maxquant peptides.txt file with Geneious Prime software (version 2021.01). Grey bars represent peptides, annotations indicate the respective AP-MS experiments (A151R or MGF360-21R) with the replicate number as suffix. MS/MS spectra representing the two peptides highlighted by an arrow which were only identified in a single replicate of the MGF360-21R pull-down experiments are shown together with the fragment pattern in panel (B). Panel (C) shows an extracted ion chromatogram (EIC, DataAnalysis software version 6.1, Bruker) for m/z 464.1924 corresponding to peptide 76-82 (DCFGCLR) which has been reliably detected in all AP-MS experiments with A151R and MGF360-21R, but not in the GFP controls. Red, blue and black tracks represent three replicates each of AP-MS experiments with MGF360-21R, A151R, and GFP controls, respectively. Retention times indicate peaks that were identified as DCFGCLR by Maxquant software after fragmentation and MS/MS analysis.
